# Influence of statin use on prognosis of patients with renal cell cancer: a meta-analysis

**DOI:** 10.3389/fonc.2023.1132177

**Published:** 2023-07-13

**Authors:** Wenli Liang, Yongmei Pan, Aixue Liu, Yan He, Yan Zhu

**Affiliations:** ^1^ Oncology Department, Shenzhen Second People’s Hospital, the First Affiliated Hospital of Shenzhen University, Shenzhen, China; ^2^ Department of Medical Imaging, the First Dongguan Affiliated Hospital of Guangdong Medical University, Dongguan, China

**Keywords:** statin, renal cell cancer, survival, prognosis, meta-analysis

## Abstract

**Background:**

Statin may confer anticancer efficacy, while the studies evaluating the influence of statin on survival of patients with renal cell cancer (RCC) yielded inconsistent results. A systematic review and meta-analysis was performed to investigate the association between statin use and survival of patients with RCC.

**Materials and Methods:**

Cohort studies were identified by search of PubMed, Embase, and Web of Science databases according to the objective of the meta-analysis. A random-effect model incorporating the possible between-study heterogeneity was used for meta-analysis. Subgroup analyses according to study characteristics were also performed.

**Results:**

Seventeen cohort studies involving 42528 patients with RCC were available for the meta-analysis. Results showed that statin use was associated with a better overall survival (OS, hazard ratio [HR]: 0.73, 95% confidence interval [CI]: 0.65 to 0.84, p < 0.001; I^2^ = 40%), progression progression-free survival (PFS, HR: 0.82, 95% CI: 0.68 to 0.98, p = 0.03; I^2^ = 52%), and cancer-specific survival (CSS, HR: 0.76, 95% CI: 0.59 to 0.99, p = 0.04; I^2^ = 38%). Besides, for the outcome of OS and PFS, subgroup analyses showed similar results in patients with surgical and non-surgical anticancer treatments, and in patients with stage I-III and stage IV RCC (p values for subgroup difference all > 0.05).

**Conclusions:**

Statin use may be associated with improved survival outcomes in patients with RCC. Although prospective clinical studies should be considered to validate these results, these findings suggest that statins may be potential adjuvant therapy for patients with RCC.

## Introduction

Renal cell cancer (RCC) is among the most common malignancy of the urinary system (1, 2). Globally, approximately 400,000 cases of RCC were diagnosed annually, and about 175,000 people died from RCC, according to the statistics in 2018 (3, 4). Besides, it could be estimated that RCC will continuously be a serious threat to the health of the global population because the worldwide incidence of RCC has been reported to be increasing continuously within the recent decades (5). While imaging techniques for cancer screening have advanced, about 30% of patients with RCC are diagnosed at an advanced stage, which may be an underlying cause to the overall poor prognosis of patients with RCC (6). Therefore, efforts are still needed to identify novel treatment options which may improve the survival of patients with RCC (7).

The statin family is a class of lipid-lowering drugs that inhibit the 3-hydroxy-3-methylglutarylcoenzyme-A reductase, a key enzyme involved in cholesterol synthesis (8). Moreover, further studies have confirmed the anti-inflammatory, anti-proliferative, pro-apoptotic, immunomodulatory, and anti-metastasizing properties of statins, suggesting that statins may inhibit the pathogenesis and progression of tumor (9–11). Accumulating evidence has suggested that statin use may be related to improved prognosis of patients with certain cancers, such as those with pancreatic cancer (12), lung cancer (13), endometrial cancer (14), and colorectal cancer (15). However, previous studies evaluating the influence of statin on survival of patients with RCC yielded inconsistent results (16). Some studies suggested that statin use may be associated with improved survival in patients with RCC (17–25), while others did not show consistent results (26–33). Therefore, in this study, we performed a systematic review and meta-analysis to comprehensively investigate the association between statin use and survival outcomes in patients with RCC.

## Materials and methods

We followed the Preferred Reporting Items for Systematic reviews and Meta-Analyses (PRISMA) Statement (34, 35) in this study. The analytic methods were in accordance with the instructions of the Cochrane’s Handbook for Systematic Review and Meta-analysis (36).

### Database search

We systematically searched the electronic databases of PubMed, Embase, and Web of Science using combined search terms including (1) “statin” OR “3-hydroxy-3-methyl-glutarylCoA reductase inhibitor” OR “CS-514” OR “simvastatin” OR “atorvastatin” OR “fluvastatin” OR “lovastatin” OR “rosuvastatin” OR “pravastatin” OR “pitavastatin”; (2) “renal” OR “kidney”; (3) “cancer” OR “tumor” OR “carcinoma” OR “neoplasm” OR “adenoma” OR “malignancy”; and (4) “recurrence” OR “death” OR “mortality” OR “survival” OR “prognosis” OR “deaths” OR “remission” OR “collapse” OR “progression” OR “metastasis”. We used filters to limit the searches to studies in humans. No restriction was applied to the language of publication. As a supplementation, we manually screened the reference lists of the related literatures for possible relevant studies. The final database search was performed on October 23, 2022.

### Study inclusion

The PICOS criteria were followed during the determination of the inclusion criteria.

P (patients): adult patients with confirmed diagnosis of RCC;

I (exposure): patients with statin use as defined by the original studies;

C (control): patients without statin use as defined by the original studies;

O (outcomes): relative risks for the incidence of overall survival (OS), progression-free survival (PFS), or cancer-specific survival (CSS) between users versus non-users of statin during follow-up durations. Specifically, OS was defined as time from diagnosis to death from any cause, PFS was defined as time from diagnosis to disease progression or relapse, unplanned re-treatment after initial management, or death from any cause, and CSS was defined as time from diagnosis to death from RCC.

S (study design): cohort studies, including the retrospective and prospective studies.

We only considered studies published as full-length articles in peer-reviewed journals. For studies with overlapped patient population, the one with the largest sample size was included. Reviews, preclinical studies, cross-sectional studies, studies did not evaluate statin use as exposure, studies including non-RCC patients, or studies did not report the survival outcomes were excluded from the meta-analysis.

### Data extracting and quality evaluation

Two authors implemented database search, data extraction, and study quality assessment separately. If disagreements occurred, they were discussed with the third author for consensus. Data regarding the study information, patient characteristics, definition of statin use, follow-up durations, and outcomes reported were collected by the two independent authors using a predefined data extraction table. The Newcastle-Ottawa Scale (NOS) (37) was used for study quality evaluation. This scale is rated from 1 to 9 stars and reflected the quality of the study by aspects of participant selection, comparability between groups, and outcome validation.

### Statistical analyses

The relative risk for the incidence of survival outcomes between users and non-users of statins were presented as hazard ratio (HR) and the corresponding 95% confidence interval (CI). For studies reported multiple HRs according to different models of multivariate regression analyses, the most adequately adjusted HR from each study was extracted and combined in this meta-analysis. Then, standard errors (SEs) of HRs were estimated from the 95% CIs or P values. For normalization of their distribution, HRs were logarithmically transformed and combined (36). Heterogeneity within the included cohort studies was tested *via* Cochrane’s Q test, as well as the estimation of I^2^ statistic (38). An I^2^ > 50% suggests significant level of heterogeneity. A random-effect model was chosen to combine the HRs by incorporating the potential heterogeneity within studies (36). Predefined subgroup analyses were conducted to explore the possible influences of study characteristics on the outcomes, including main anticancer treatment (surgical versus non-surgical), and the clinical stages of the tumor. Funnel plots were constructed, and were used for the assessment of publication bias (36). Visually asymmetrical funnel plots implied potential publication bias, which could be further validated by the Egger’s regression asymmetry test. The RevMan (Version 5.1; Cochrane Collaboration, Oxford, UK) and Stata (version 12.0; Stata Corporation, College Station, TX) software was used for the statistical analyses.

## Results

### Literature search

**Figure 1:**
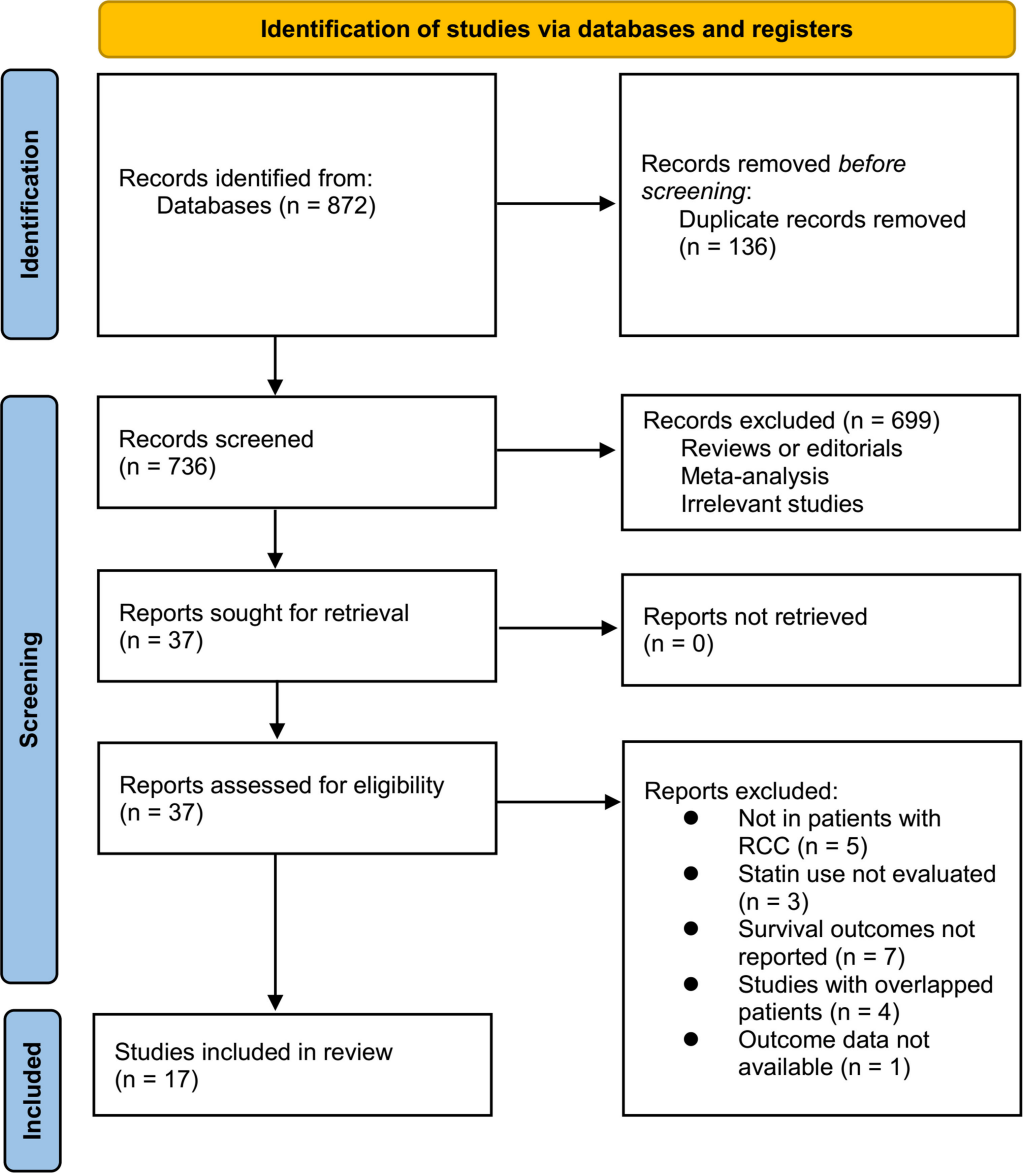
PRISMA diagram of literature search and study inclusion.

**Figure 1** summarizes the process of literature search. In brief, 872 articles were retrieved in initial database search, and 736 articles were obtained after excluding the duplications. Then, 37 articles were considered to be potentially relevant after excluding 699 irrelevant articles in title and abstract screening. Through full-text review, anther 20 studies were further excluded because of the reasons listed in **Figure 1**. Finally, 17 cohort studies were included in the meta-analysis (17–33).

### Summary of study characteristics

Seventeen cohort studies (17–33) involving 42528 patients with RCC were available for the meta-analysis. The characteristics of the included studies are shown in **Table 1**. Briefly, these studies were published between 2012 and 2022, and performed in Australia, Denmark, Korea, the United States, Canada, Germany, Austria, Czech, Italy, and Spain. All of the studies were retrospective cohort studies except for one study, which was a prospective study (31). All the patients were diagnosed with RCC. The mean ages of the patients were 57 to 67 years, and the proportions of men were 56 to 75%. In ten of the included studies (18–21, 24, 27–30, 32), surgeries such as nephrectomy were performed, while in five studies (17, 22, 25, 31, 33), non-surgical therapies such as interferon, temsirolimus, Immunotherapy, and target drugs were applied. In 15 of the included studies, statin use was defined as confirmed concurrent use of statin *via* medical charts at the diagnosis of RCC (17–25, 27, 29–33), while for the other two studies (26, 28), stain use was defined as regular statin use before the diagnosis of RCC. Accordingly, 10123 (23.8%) patients were statin users. The mean follow-up durations varied from 12 to 94 months, and confounding factors such as age, sex, tumor stage, performance status, and comorbidities etc. were controlled in the multivariate analyses. The NOS for the studies varied between six and nine, indicating good study quality (**Table 2**).

**Table 1:** Characteristics of the included studies.

Study	Design	Country	No. of patients	Mean age (years)	Men (%)	Main treatment	Stage	Definition of statin use	Number of patients with statin use	Median follow-up durations (months)	Variables adjusted	Outcomes reported
Lee 2012	RC	Australia	416	59	69	Interferon and temsirolimus	IV	Concurrent statin use as evidenced by medical records	34	17.9	Age, sex, geographic region, nephrectomy status, histological type, KPS, and levels of HGB, LDH, and corrected serum calcium.	OS and PFS
Nielsen 2012	RC	Denmark	1152	69	56	NR	I-IV	Regular statin use before the diagnosis of RCC	384	31.2	Age, sex, tumor stage, and treatments	CSS
Choi 2013	RC	Korea	115	61.3	62.6	Radical or partial nephrectomy	I-III	Concurrent statin use as evidenced by medical records	21	40	Age, sex, and BMI	PFS
Krane 2014	RC	USA	339	61.8	NR	Robot-assisted partial nephrectomy	I-III	Concurrent statin use as evidenced by medical records	104	19.7	Age, sex, comorbidities, and tumor stage	PFS
Hamilton 2014	RC	USA	2608	61.2	65.1	Surgical resection	I-IV	Concurrent statin use as evidenced by medical records	699	36	Age, sex, ethnicity, surgery type, CCI, tumor stage, eGFR,	OS and PFS
Viers 2015	RC	USA	2357	63	67	Nephrectomy	I-III	Regular statin use within 3 months before the diagnosis of RCC	630	93.6	Age, sex, smoking, KPS, CCI, surgery type, BMI, tumor size, tumor stage, and histological type	OS, CSS, and PFS
Kaffenberger 2015	RC	USA	916	60.8	65	Radical or partial nephrectomy	I-IV	Concurrent statin use as evidenced by medical records	270	42.5	Age, sex, ASA class, tumor stage, grade, corrected hypercalcemia, and anemia	OS and CSS
Haddad 2015	RC	USA	850	57.1	57.9	Surgical resection	I-III	Concurrent statin use as evidenced by medical records	342	25	Age, sex, BMI, race, ECOG PS, tumor grade, histological type, LVI, SCr, and surgery type	OS and PFS
McKay 2016	RC	USA	4736	NR	71.2	Target therapy	IV	Concurrent statin use as evidenced by medical records	511	30	Age, sex, race, histology, prior therapy, sites of metastasis, IMDC risk factors, baseline dyslipidemia and BMI	OS and PFS
Nayan 2016	RC	Canada	893	57.4	64.3	Nephrectomy	I-III	Concurrent statin use as evidenced by medical records	259	47	Age, sex, CCI, type of surgery, tumor stage, and histology	OS, CSS, and PFS
El-Refai 2017	RC	USA	26107	63.2	53.6	NR	I-IV	Concurrent statin use as evidenced by medical records	6308	12	Age, sex, CCI, and concurrent medications	OS
Berquist 2017	RC	USA	283	57.5	66.1	Nephrectomy	I-III	Concurrent statin use as evidenced by medical records	180	68	Age, sex, BMI, race, ASA class, cancer stage, histologic grade, and surgery type	OS, CSS, and PFS
Neumann 2019	RC	Germany	388	64.2	66.8	Nephrectomy	I-III	Concurrent statin use as evidenced by medical records	39	57.9	Age, sex, smoking, tumor stage, grade, histological type, and Ki67	OS
Boegemann 2020	PC	Germany	557	67	71.8	Target therapy	IV	Concurrent statin use as evidenced by medical records	130	40	Age, sex, BMI, tumor grade, and IMDC risk factors	OS and PFS
Heide 2020	RC	Austria and USA	164	62	66	Nephrectomy	I-III	Concurrent statin use as evidenced by medical records	41	35.4	Age, sex, tumor stage, grade, and surgery type	OS
Fiala 2021	RC	Czech	343	64.5	74.3	Target therapy	IV	Concurrent statin use as evidenced by medical records	78	19.9	Age, sex, BMI, and tumor grade	OS and PFS
Santoni 2022	RC	Italy, Spain and the USA	304	NR	74	Immunotherapy and/or target therapy	IV	Concurrent statin use as evidenced by medical records	93	35.8	Age, sex, previous nephrectomy, histology type, and IMDC risk factors	OS and PFS

RC, retrospective cohort; PC, prospective cohort; NR, not reported; KPS, Karnofsky Performance Scale; LDH, lactate dehydrogenase; HGB, hemoglobin; BMI, body mass index; CCI, Charlson Comorbidity Index; eGFR, estimated glomerular filtrating rate; ASA, American Society of Anesthesia; ECOG PS, Eastern Cooperative Oncology Group Performance Status; LVI, lymphovascular invasion; SCr, serum creatinine; IMDC, International Metastatic RCC Database Consortium; OS, overall survival; PFS, progression-free survival; CSS, cancer-specific survival.

**Table 2:** Study quality evaluation *via* the Newcastle-Ottawa Scale.

Study	Representativeness of the exposed cohort	Selection of the non-exposed cohort	Ascertainment of exposure	Outcome not present at baseline	Control for age and sex	Control for other confounding factors	Assessment of outcome	Enough long follow-up duration	Adequacy of follow-up of cohorts	Total
Lee 2012	0	1	1	1	1	1	1	1	1	8
Nielsen 2012	0	1	1	1	1	1	1	1	1	8
Choi 2013	0	1	1	1	1	0	1	1	1	7
Krane 2014	0	1	1	1	1	1	1	1	1	8
Hamilton 2014	0	1	1	1	1	1	1	1	1	8
Viers 2015	0	1	1	1	1	1	1	1	1	8
Kaffenberger 2015	0	1	1	1	1	1	1	1	1	8
Haddad 2015	0	1	1	1	1	1	1	1	1	8
McKay 2016	0	1	1	1	1	1	1	1	1	8
Nayan 2016	0	1	1	1	1	1	1	1	1	8
El-Refai 2017	0	1	1	1	1	0	1	0	1	6
Berquist 2017	0	1	1	1	1	1	1	1	1	8
Neumann 2019	0	1	1	1	1	1	1	1	1	8
Boegemann 2020	1	1	1	1	1	1	1	1	1	9
Heide 2020	0	1	1	1	1	1	1	1	1	8
Fiala 2021	1	1	1	1	1	1	1	1	1	9
Santoni 2022	0	1	1	1	1	1	1	1	1	8

### Association between statin use and OS of patients with RCC

**Figure 2:**
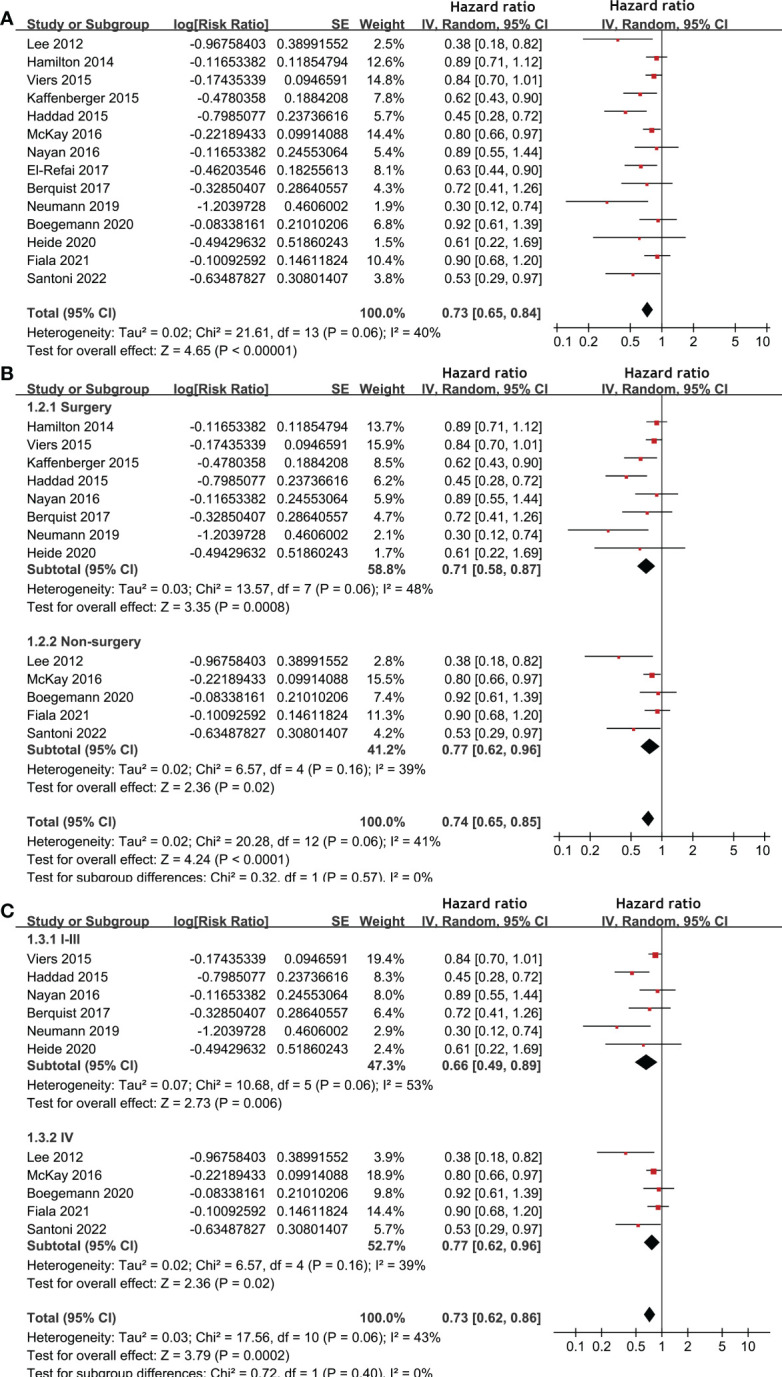
Forest plots for the meta-analysis of the association between statin use and OS of patients with RCC. **(A)**, overall meta-analysis; **(B)**, subgroup analysis according to the main treatment; and **(C)**, subgroup analysis according to the tumor stage.

Pooled results of 14 studies (17, 18, 20–25, 28–33) showed that statin use was associated with a better OS (HR: 0.73, 95% CI: 0.65 to 0.84, p < 0.001; I^2^ = 40%; **Figure 2A**) of patients with RCC. Subgroup analyses showed similar results in patients with surgical (HR: 0.71, 95% CI: 0.58 to 0.87, p < 0.001; I^2^ = 48%) and non-surgical anticancer treatments (HR: 0.74, 95% CI: 0.65 to 0.85, p < 0.001; I^2^ = 41%; p for subgroup difference = 0.57; **Figure 2B**), and in patients with stage I-III (HR: 0.66, 95% CI: 0.49 to 0.89, p = 0.006; I^2^ = 53%) and stage IV RCC (HR: 0.77, 95% CI: 0.62 to 0.96, p < 0.02; I^2^ = 39%; p for subgroup difference = 0.40; **Figure 2C**).

### Influence of statin use on PFS and CCS in patients with RCC

**Figure 3:**
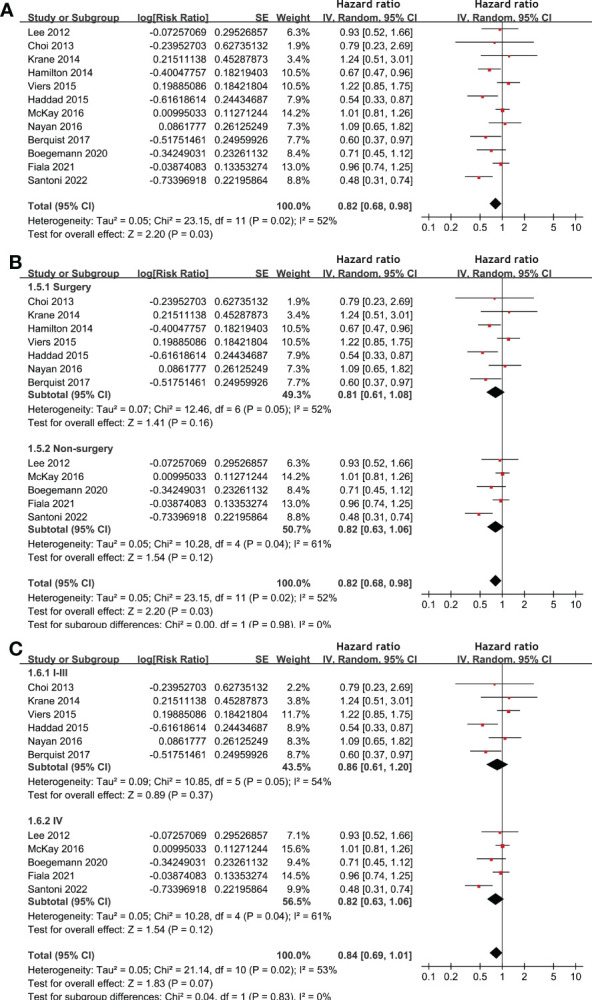
Forest plots for the meta-analysis of the association between statin use and PFS of patients with RCC. **(A)**, overall meta-analysis; **(B)**, subgroup analysis according to the main treatment; and **(C)**, subgroup analysis according to the tumor stage.

**Figure 4:**
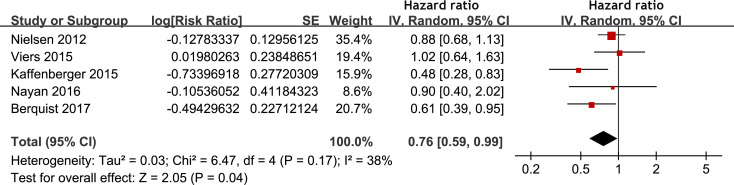
Forest plots for the meta-analysis of the association between statin use and CSS of patients with RCC.

Meta-analysis of 12 studies (17–20, 22, 25, 27–31, 33) indicated that statin use was associated with an improved PFS of patients with RCC (HR: 0.82, 95% CI: 0.68 to 0.98, p = 0.03; I^2^ = 52%; **Figure 3A**). Subgroup analyses showed similar results in patients with surgical and non-surgical anticancer treatments (p for subgroup difference = 0.98, **Figure 3B**), and in patients with stage I-III and stage IV RCC (p for subgroup difference = 0.83, **Figure 3C**). In addition, pooling the results of five studies (21, 26, 28–30) suggested that statin use was associated with an improved CSS (HR: 0.76, 95% CI: 0.59 to 0.99, p = 0.04; I^2^ = 38%; **Figure 4**) in patients with RCC.

### Publication bias

**Figure 5:**
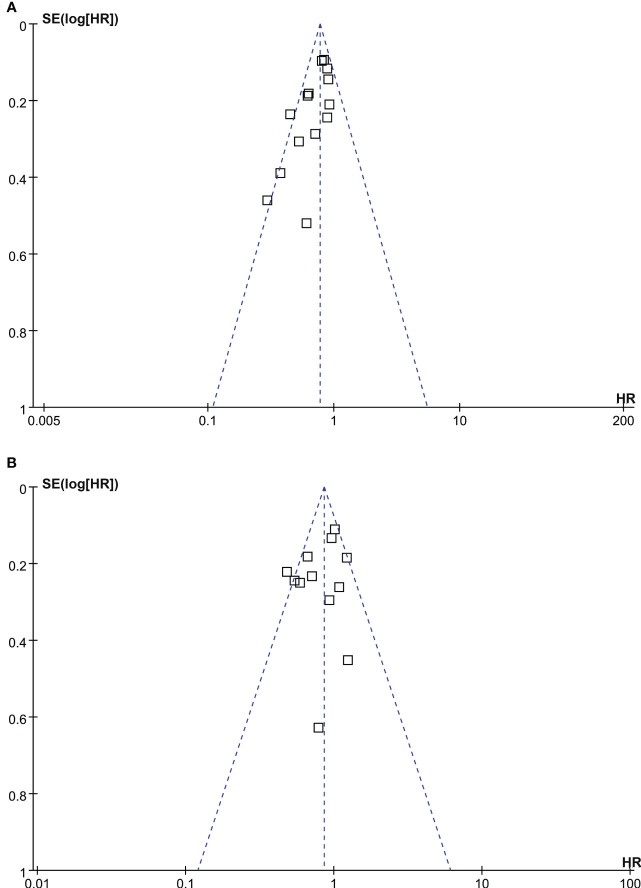
Funnel plots for the meta-analyses; **(A)**, funnel plots for the association between statin use and OS of patients with RCC; and **(B)**, funnel plots for the association between statin use and PFS of patients with RCC.

Funnel plots for the meta-analyses of OS and PFS were symmetrical on visual examination (**Figures 5A, B** **)**, suggesting low risk of publication biases. Egger’s regression tests showed consistent results (p = 0.17 and 0.33, respectively). The publication bias for the meta-analysis of CSS was unable to determine because only five studies were included for the outcome.

## Discussion

In this study, by pooling the results of 17 available cohort studies, results of the meta-analysis showed that compared non-users of statin, RCC patients who used statin were associated with improved survival outcomes, including OS, PFS, and CSS. In addition, subgroup analyses showed consistent results in patients who were treated surgically and non-surgically, and in patients with stage I-III and stage IV RCC. Collectively, these findings suggest that statin use may be associated with an improved survival in patients with RCC.

To the best of our knowledge, there are three meta-analyses which evaluated the association between statin use and prognosis in patients with RCC. An early meta-analysis published in 2015 showed that statin use may improve the OS in patients with RCC, while other outcomes, such as PFS or CSS were not significantly affected (39). The authors therefore concluded that although a benefit of statin on survival was suggested, this may not be related to the anticancer efficacy of statin because outcomes related to the tumor progression was not significantly affected (39). However, only four cohort studies were included in the meta-analysis, which made the results less convincing (39). A subsequent meta-analysis in 2017 with 12 studies suggested that statin use in patients with RCC was associated with improved OS and CSS, but not PFS. However, for the outcome of PFS, only two studies were available, which made the results also less reliable (40). A recent meta-analysis with literatures by July 2019 showed that statin use in patients with RCC was not associated with improved OS (41). However, the process of literature searching in this meta-analysis may have flaw because only 5 studies were included, and considerable eligible studies were not enrolled (41). Collectively, the influence of statin on survival of patients with RCC remains not fully determined to date. Our meta-analysis has several strengths in methodology as compared to the previous ones. Firstly, we performed updated literature search in three commonly used electronic databases, and retrieved 17 up-to-date eligible studies. Among them, six were published recently and not included in the previous meta-analyses (23–25, 31–33). Secondly, only cohort studies were included, which could suggest a longitudinal relationship between statin use and improved survival in patients with RCC. Also, multivariate analyses were performed in all available studies when the association between statin and survival of patients with RCC was estimated, which minimized the potential influence of confounding factors. Moreover, three commonly used survival outcomes including OS, PFS, and CSS were all analyzed in this study, and the consistent results confirmed the robustness of the findings that statin may attenuate the progression of RCC. Finally, the relative large number of included studies enabled us to perform subgroup analyses according to the anticancer treatments and tumor stages of RCC. The consistent results of these subgroup analyses further validated the stability of the findings. Taken together, results of this meta-analysis indicated that statin use may be associated with improved survival outcomes in patients with RCC, which were independent of the anticancer treatment and the stage of the tumor.

The potential mechanisms for the improved survival of statin users with RCC may be multifactorial. Early *in vitro* studies showed that by causing cell cycle arrest and apoptosis, simvastatin inhibited the growth of RCC cells in a dose- and time-dependent manner, when cholesterol was depleted and prenylation-associated mechanisms were involved (42). In addition, fluvastatin was demonstrated to enhance the phosphorylation of AKT, mammalian target of rapamycin, and extracellular signal-regulated kinase, resulting in a reduction in the movement of RCC cells *in vitro* (43). In addition, in combination with sorafenib, a vascular endothelial growth factor inhibitor, lovastatin showed synergistic effects against RCC cell lines proliferation (44). Finally, a recent study suggested that simvastatin could inhibit RCC cell viability, migration, invasion, and regulated the cell cycle and induced apoptosis, which were associated with the restoration of the abnormal expression of DDX5/DUSP5 in RCC. Further studies are needed to determine the major molecular mechanisms and signaling pathways underlying the potential anticancer efficacy of statins for RCC.

Our study has limitations. Firstly, most of the included studies are retrospective, which may be associated with the risks of recall and selection biases. Therefore, results of the study should be validated in large-scale prospective studies. In addition, due to the insufficient data of the included studies, we were unable to determine if some study characteristics may affect the outcomes, such as the histological type of RCC, ethnicity and sex of the patients, and type, dose, and treatment duration of statins. These factors may lead to the between study heterogeneity of the meta-analysis. Moreover, although multivariate analysis was applied among the included studies when the associations between statin use and survival outcomes of patients with RCC were estimated, we could not exclude the possibility that there were still residual factors which may confound the results, such as the cholesterol levels of the patients. Finally, a causative relationship between statin use and improved survival of patients with RCC could not be established on the basis of our finding, because this meta-analysis was based on the results of observational studies. Clinical trials should be performed to evaluate the role of statins as adjuvant treatment for patients with RCC.

In conclusion, statin use may be associated with improved survival outcomes in patients with RCC. Although prospective clinical studies should be considered to validate these results, these findings suggest that statins may be potential adjuvant therapy for patients with RCC.

## Data availability statement

The original contributions presented in the study are included in the article/supplementary material. Further inquiries can be directed to the corresponding author.

## Author contributions

WL conceived the study. WL and YP performed database search, literature review, study selection, quality evaluation, and data collection. WL, AL, YH, and YZ performed data statistics and interpreted the results. WL drafted the manuscript. All authors contributed to the article and approved the submitted version.
